# The Prevalence of STIV c92-Like Proteins in Acidic Thermal Environments

**DOI:** 10.1155/2011/650930

**Published:** 2011-07-14

**Authors:** Jamie C. Snyder, Benjamin Bolduc, Mary M. Bateson, Mark J. Young

**Affiliations:** ^1^Department of Plant Sciences and Plant Pathology, Montana State University, Bozeman, MT 59717, USA; ^2^Thermal Biology Institute, Montana State University, Bozeman, MT 59717, USA; ^3^Department of Chemistry and Biochemistry, Montana State University, Bozeman, MT 59717, USA

## Abstract

A new type of viral-induced lysis system has recently been discovered for two unrelated archaeal viruses, STIV and SIRV2. Prior to the lysis of the infected host cell, unique pyramid-like lysis structures are formed on the cell surface by the protrusion of the underlying cell membrane through the overlying external S-layer. It is through these pyramid structures that assembled virions are released during lysis. The STIV viral protein c92 is responsible for the formation of these lysis structures. We searched for c92-like proteins in viral sequences present in multiple viral and cellular metagenomic libraries from Yellowstone National Park acidic hot spring environments. Phylogenetic analysis of these proteins demonstrates that, although c92-like proteins are detected in these environments, some are quite divergent and may represent new viral families. We hypothesize that this new viral lysis system is common within diverse archaeal viral populations found within acidic hot springs.

## 1. Introduction

Compared to the viruses infecting organisms from the domains *Bacteria* and *Eukarya*, few viruses infecting archaeal organisms have been isolated and most are poorly understood in molecular detail. Most archaeal viruses are morphologically and genetically distinct from previously described viruses [1–4]. *Sulfolobus* turreted icosahedral virus (STIV) has emerged as a model system for examining archaeal virus replication and structure [5–15]. STIV was originally isolated from the Rabbit Creek thermal basin within Yellowstone National Park, (YNP) USA [15]. The STIV virion contains a circular double-stranded (ds) DNA genome of 17,663 base pairs (bps) which encodes for 37 open reading frames (ORFs). The cryoelectron microscopy image reconstruction of STIV particles revealed a pseudo *T* = 31 capsid, an internal membrane, and turret-like projections extending from each of the virions fivefold axes [15]. Structural analysis of the major capsid protein has suggested an evolutionary link between archaeal viruses and bacterial and eukaryotic viruses [7, 15]. 

**Figure 1:**
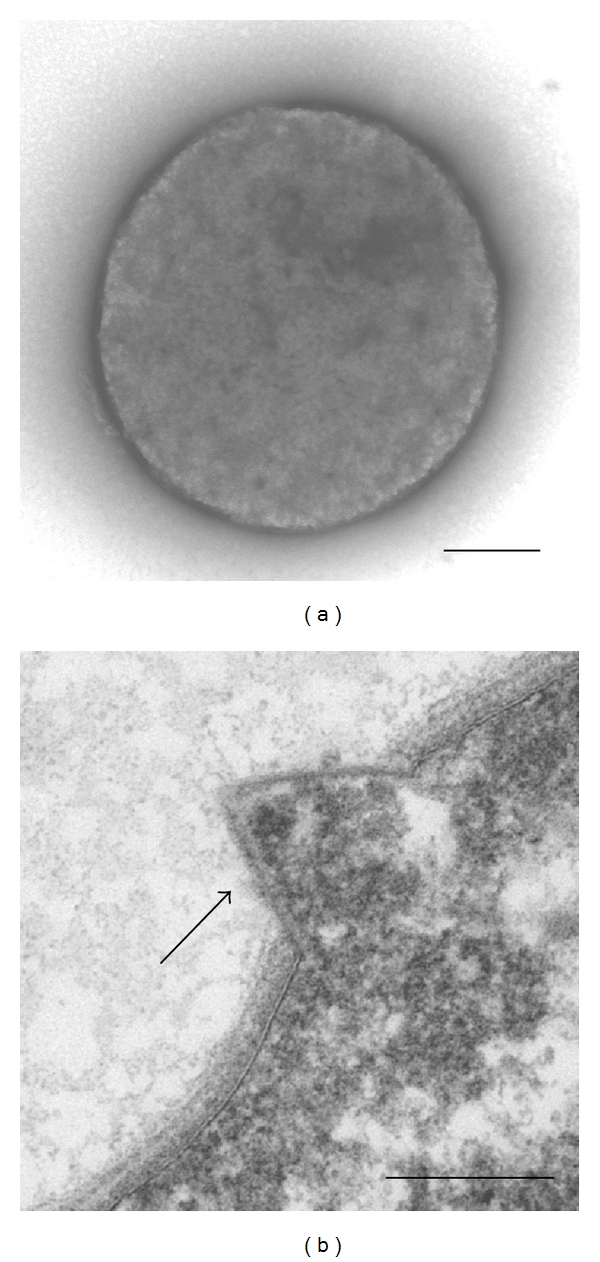
Electron micrographs of *Sulfolobus* cells (a) not infected with STIV and (b) infected with STIV; arrow indicates the STIV-induced pyramid lysis structure. Bars: 250 nm (a) and 100 nm (b).

Many viruses have evolved specific mechanisms for exiting their host cell at the end of their viral replication cycle. Lytic viruses of bacteria typically utilize a holin/endolysin-based mechanism for virion release [16, 17], whereas virion egress via a cellular budding mechanism is commonly used by enveloped animal viruses. A new lysis system was recently described for STIV [12, 14]. This lysis system involves the formation of unique pyramid lysis structures on the membrane of an infected cell prior to lysis (Figure 1). The lysis structures are a result of the protrusion of the cellular membrane through the S-layer surrounding the cell. A similar lysis system was more recently described in another archaeal virus, *Sulfolobus islandicus* rod-shaped virus 2 (SIRV2) [18–20], indicating that this type of lysis may be widespread among archaeal viruses. Even though these two different archaeal viruses appear to share a similar lysis mechanism, genetically they are very different from each other. Only one ORF shows any similarity between the two archaeal viruses [19]. A homologous viral protein from STIV and SIRV2 was recently identified as being responsible for the formation of the pyramid lysis structures on the cell membrane [14, 18]. The STIV protein c92 [14] and the SIRV2 protein p98 [18] were independently heterologously overexpressed in *Sulfolobus* cells. Both proteins resulted in the formation of pyramid lysis structures in the absence of any other viral proteins. While the overexpression of the STIV c92 protein produced the pyramid-like structures, it did not directly lead to cell lysis, indicating there must be additional factors that are required to complete lysis of the host cell.

We are interested in examining the extent that c92-like lysis systems operate in crenarchaeal virus populations present in natural high-temperature low-pH environments. To determine whether this type of lysis system is common in crenarchaeal viruses, we compared the STIV c92 protein to archaeal cellular and viral metagenomic databases that we have produced from multiple acidic hot springs located in YNP. From this analysis, we conclude that c92-like proteins are present in the natural environment and lytic crenarchaeal viruses may be more prevalent than once thought.

## 2. Methods

Numerous metagenomic libraries from two geographically isolated thermal areas within YNP were used in this study. The first area is the Crater Hills thermal basin, containing an acidic (pH ~ 2.5) high-temperature (~82°C) hot spring (44.6532°N, 110.4847°W). The second thermal site is in the Nymph Lake area (44.7536°N, 110.7237°W) within Norris Geyser Basin in YNP and also contains acidic (pH ~ 3.5–4.5) and high-temperature (~91°C) hot springs. Over the course of four years we have produced a total of 39 metagenomic libraries from these two thermal areas within YNP. The metagenomes are enriched for either viral or cellular sequences by methods previously described ([21], B. Bolduc, F. F. Roberto, M. Lavin, and M. J. Young, manuscript in prep.).

The c92 protein encoded by STIV and its homologues found in a number of rudiviruses (SIRV2, SIRV1 variant XX, and SIRV1 [19]) were used to find related proteins using tBLASTn (http://blast.ncbi.nlm.nih.gov/) in a collection of 39 viral and cellular metagenomes produced from YNP acidic hot springs that contained more than 2.8 million sequencing reads and 807 megabases (Mb) of DNA sequence. An alignment of identified protein sequences from the YNP metagenomes and related proteins found in other archaeal viruses was performed using ClustalW (Blosum cost matrix) [22]. Following the alignment, phylogenetic analysis was executed using Mr. Bayes for 1.5 million generations, with a burn-in of 10,000 trees and sampling every 10,000 trees [23].

## 3. Results

The communities present in the acidic hot springs within YNP are dominated by archaeal organisms [21, 24, 25]. The unique viral lysis system described for STIV [12, 14] and SIRV2 [18–20] appears within the archaeal viral community associated within YNP acidic hot springs. By comparing STIV c92 and homologues in rudiviral genomes against our YNP viral and cellular metagenomic databases, we found 70 contigs coding for protein sequences that represent closely related proteins.

A tBLASTn search identified 70 contig matches to the STIV c92 protein. Alignment lengths varied from 36 to 92 (c92 full length) amino acid residues. The vast majority of matches were significant with *e*-values <10^−7^ Most of the 70 contigs with matches to c92 from the metagenomic database (58 contigs representing 83% of the total number of c92 matches) aligned to at least 60 residues of the STIV c92 protein based on inferred amino acid similarity. Meanwhile, other sequences (12 contigs representing 17% of the total number of c92 matches) only aligned to less than half of the c92 sequence, because the end of the assembled contig fell within the c92-like sequence. The sequence variability within the c92 protein was evenly spread through the entire length of the sequence. However, the N-terminal domains of the c92-like proteins consistently show a membrane spanning helix.

**Figure 2:**
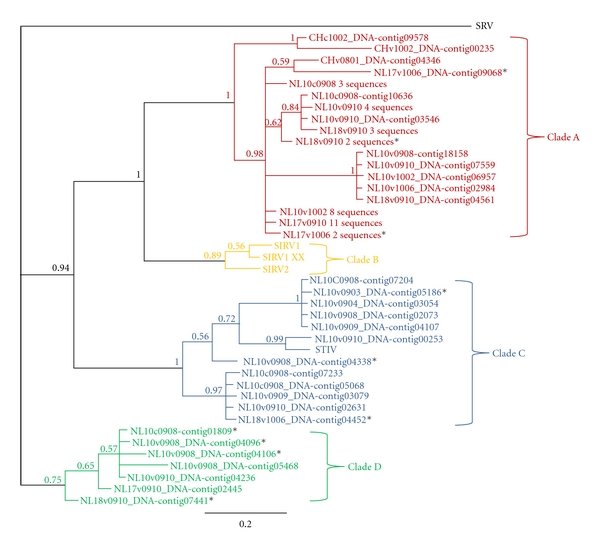
A maximum likelihood phylogenetic tree of c92-like proteins from YNP metagenomes illustrating three major families. SRV: *Stygiolobus* rod-shaped virus (outgroup); SIRV1: *Sulfolobus islandicus* rod-shaped virus 1; SIRV1 XX: SIRV1 variant XX; SIRV2: *Sulfolobus islandicus* rod-shaped virus 2; STIV: *Sulfolobus* turreted icosahedral virus; NL: Nymph Lake metagenomes; CH: Crater Hills metagenomes; sample names indicate cellular (c) or viral (v) metagenomes and the year followed by the month of sample collection; short contigs indicated by *. The numbers at the branches represent clade probabilities, and the scale bar represents the average number of substitutions per site.

Phylogenetic analyses were performed on all contigs that aligned to c92 and on an alignment of the 58 contigs with complete c92-like proteins (Figure 2; contigs with incomplete c92-like proteins are indicated by *). Three well-supported clades of c92-like proteins were identified in the metagenomic data from Crater Hills and Nymph Lake (Figure 2). The SIRV family (Figure 2; Clade B) does not appear to have close relatives in our metagenomes. While it is likely that many of the sequences with near 100% amino acid similarity to the c92 protein represent STIV-like viruses known to exist in these environments (Figure 2; Clade C), others are more divergent representing the “c92-like” family of proteins and are present in viruses unrelated to STIV. There are two groups of contigs that are distinct from STIV and rudiviruses but, they comprise the vast majority of all the c92 matches (Figure 2; Clades A and D). The sequences with c92 matches belonging to Clade A comprise the largest group of c92-like proteins represented in our metagenomes (1,153 reads); however, all of the clades contained similar average contig lengths (Table 1). 

**Figure 3:**
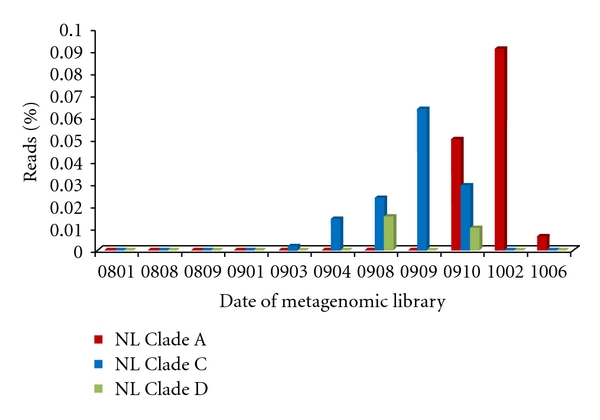
The temporal dynamics of c92-like protein clades in viral metagenomic data sets from a Nymph Lake (NL10) hot spring.

The characteristics of the metagenomes with matches to c92 are shown in Table 2. The contigs that matched c92 were primarily from viral DNA metagenomes created from the Nymph Lake hot springs (Table 2). For one of the Nymph Lake hot springs (NL10), the sampling dates for the libraries span from August 2008 through June 2010. The c92-like proteins from Clade A were detected in metagenomes from October 2009 through June 2010, while Clade C types were detected only from March 2009 through October 2009 (Figure 3). Clade D contained c92-like proteins that were present in August 2009, not detected in September 2009, then detected again in October 2009 (Figure 3). For the Crater Hills hot spring, the sampling dates for the libraries span from April 2007 through February 2010. The c92-like proteins from Crater Hills all belonged to Clade A but were detected only two of the five Crater Hills metagenomes (January 2008 and February 2010).

## 4. Discussion

Little is known about the viruses that infect organisms from the domain *Archaea*. Previously, it had been suggested that most archaeal viruses were nonlytic primarily due to the harsh conditions that many of these organisms inhabit and to the presence of integrase-like genes in many of the viral genomes. STIV was the first lytic crenarchaeal virus discovered [13], and SIRV2 was subsequently found to be lytic as well [20]. Homologous viral proteins from STIV and SIRV2 were discovered to be responsible for the formation of the unique pyramid lysis structures present on the *Sulfolobus* cellular membrane prior to viral-induced lysis [14, 18]. The presence of the viral-induced pyramid-like lysis structures on two unrelated viruses led us to investigate the prevalence of this type of viral lysis system in *Archaea*. We sought to determine the presence of c92-like proteins present in metagenomic datasets by comparing the sequenced STIV c92 protein to sequences collected from YNP.

Overall, there were three major clades of c92-like proteins detected in metagenomes from YNP (Figure 2). It surprised us that we did not detect relatives of the rudiviral clade (Figure 2; Clade B) in the YNP metagenomes even though we commonly detect SIRV-like viruses and sequences in acidic hot springs within YNP [21, 24, 26]. The majority of the c92-like sequences are in clades that are distantly related to STIV or SIRV2 (Figure 2; Clades A and D) and may represent new families of viruses that use a similar lysis mechanism. The clade that contains STIV (Figure 2; Clade C) contains sequences only from the Nymph Lake hot springs. The majority of c92-like protein sequences were detected in the Nymph Lake viral DNA metagenomes (Table 2). However, we did detect few c92-like sequences in the cellular metagenomes, which likely represent intracellular viruses.

It surprised us that we detected few c92-like sequences in Crater Hills compared to the number of c92-like sequences detected in Nymph Lake. The reason for this result is currently not understood; however, we speculate that differences in the water chemistry between these two sites may have an influence. The Crater Hills hot spring is a high-chloride, high-sulfur hot spring as compared to the Nymph Lake hot spring. The differences in water geochemistry, along with pH and temperature, may play a role in defining the microbial community composition and therefore virus community composition. 

The alignments of the c92-like proteins revealed variation throughout the protein sequence. The c92-like proteins can be quite different and still function to form pyramid structures as is shown by the STIV c92 and SIRV2 p98 proteins. These homologous proteins are only 55.4% identical at the amino acid level, but both result in the formation of pyramid structures [14, 18]. The N-terminal domain of both STIV c92 and SIRV p98 is a predicted membrane spanning helix [19]. Secondary structure predictions of the c92-like proteins from the metagenomes also reveal a membrane spanning helix. Current experiments are underway to explore what residues in the STIV c92 protein are critical for the formation of the pyramid lysis structures.

The detection of c92-like proteins in our metagenomic data exhibits a dynamic pattern. Temporal analysis of the Nymph Lake metagenomic data reveals at times these c92-like proteins are undetectable, while at other times they are persistent (although the clade that is present fluctuates) in the hot springs (Figure 3). This dynamic pattern has previously been shown for viruses within YNP [24]. 

This lysis system is not universal. STIV c92 homologues are not evident in the ~48 other archaeal viruses sequenced to date. Even other viruses isolated from *Sulfolobus* species do not have homologues to STIV c92 and/or SIRV2 p98 [19, 27]. This suggests that viruses in the same family may have evolved independent mechanisms to accomplish progeny virion release from infected cells. We are now challenged to isolate and further characterize archaeal viruses with phylogenetic relationships to the c92 family of proteins to confirm that they undergo host lysis mechanisms similar to STIV and SIRV2.

## Figures and Tables

**Table 1:** Sequence statistics for the three clades of c92-like proteins present in YNP.

	No. of reads	Average no. of reads/contig	Average contig length
Clade A	1153	64	592
Clade C	259	22	615
Clade D	98	14	583

**Table 2:** Characteristics of the c92 matches to YNP metagenomes.

	Total Matches	DNA viral libraries	RNA viral libraries	Cellular libraries
Crater Hills	3 (4%)^1^	2 (3%)	0 (0%)	1 (1%)
Nymph Lake	67 (96%)	56 (80%)	2 (3%)	9 (13%)

Total	70 (100%)	58 (83%)	2 (3%)	10 (14%)

^1^Indicates percentage of the total number of contigs (70) detected.
